# Muscle Fatigue in Dynamic Movement: Limitations and Challenges, Experimental Design, and New Research Horizons

**DOI:** 10.3390/bioengineering13020248

**Published:** 2026-02-20

**Authors:** Natalia Daniel, Jerzy Małachowski, Kamil Sybilski, Michalina Błażkiewicz

**Affiliations:** 1 Institute of Rocket Technology and Mechatronics, Faculty of Mechatronics, Armament and Aviation, Military University of Technology, 00-908 Warsaw, Poland; natalia.daniel@wat.edu.pl; 2 Institute of Mechanics & Computational Engineering, Faculty of Mechanical Engineering, Military University of Technology, 00-908 Warsaw, Poland; kamil.sybilski@wat.edu.pl; 3 Faculty of Rehabilitation, Józef Piłsudski University of Physical Education in Warsaw, 00-968 Warsaw, Poland; michalina.blazkiewicz@awf.edu.pl

**Keywords:** EMG, DWT, AI, dynamic movement

## Abstract

Research on muscle fatigue during dynamic movement using surface electromyography (sEMG) constitutes a significant challenge within biomechanics. Despite a degree of standardization, measurements and their resultant findings continue to attract considerable debate, attributable to factors such as skin impedance, perspiration, and electrode displacement, as well as subjective fatigue perception. Further questions remain regarding signal normalization and the selection of appropriate analytical methodologies. Recent years have witnessed notable progress in dynamic fatigue research, highlighting the limitations of classical metrics (e.g., EMG Median Frequency) and introducing time–frequency methods, such as the wavelet transform (WT), which are better equipped to handle signal non-stationarity. Interest has also expanded to include non-linear metrics (e.g., entropy) and the analysis of multiple signals (EMG, accelerometers, fNIRS, EEG). The inherent complexity of conducting studies under conditions that approximate real-world sporting disciplines requires the consideration of the influence of various confounding factors. The judicious selection of relevant physical activities and the rigorous validation of the measurement apparatus are paramount for the accurate execution of the calculations. Current research is substantially predicated on artificial intelligence (AI) algorithms. The synergistic application of AI with wavelet transform, particularly in the decomposition and extraction of EMG signals, demonstrates efficacy in fatigue detection. Nevertheless, the full realization of these potential mandates requires further investigation into system generalization, the integration of data from multiple sensors, and the standardization of protocols, coupled with the establishment of publicly accessible datasets. This article delineates selected guidelines and challenges pertinent to the planning and execution of research on muscle fatigue in dynamic movement, focusing on activity selection, equipment validation, EMG signal analysis, and AI utilization.

## 1. Introduction

Investigating muscle fatigue during dynamic movement remains a formidable challenge in biomechanics. Although surface electromyography (sEMG) has been used for decades to quantify muscle activity and fatigue [1,2], and despite efforts to standardize these procedures [3,4,5], the optimal methodological approach and interpretation of results remain subjects of significant debate [6]. Recently, numerous guidelines have emerged that aim to mitigate the limitations frequently encountered by researchers [7,8,9]. Critical confounding factors include the impedance of the electrode-skin, which compromises the signal fidelity [10], perspiration, motion artifacts (e.g., electrode displacement or detachment), and the anatomical variability of electrode placement sites [11]. These technical challenges are further compounded by physiological variables, such as subjective fatigue perception [12]. From a signal processing perspective, researchers face persistent questions regarding signal normalization strategies [13], the selection of appropriate computational methods, and the standardization of protocols across different sports activities [14].

Significant progress has recently been made in characterizing muscle fatigue during dynamic tasks. It is currently well established that classical spectral indices, such as Median Frequency (MDF) or Mean Frequency (MNF), provide useful but ultimately insufficient information for dynamic conditions. To address the inherent non-stationarity of these signals, time–frequency methods—including the Wavelet Transform (WT)—and adaptive techniques such as Empirical Mode Decomposition (EMD) and the Hilbert–Huang Transform (HHT) have been implemented to facilitate continuous fatigue monitoring [15,16]. Wavelet-based analyses have demonstrated superior stability compared to the Fast Fourier Transform (FFT) in long-duration dynamic experiments [15]. Despite these advantages, alternative methods continue to be explored. Interest has surged in non-linear metrics, such as entropy [17], and parameter assessment based on multiple signals, the combination of EMG measurements with accelerometers [18], Functional Near-Infrared Spectroscopy (fNIRS) or Electroencephalography (EEG) [19,20]. This methodological complexity offers opportunities to evaluate fatigue under conditions that approximate real-world performance, including the emerging study of fatigue within simulated Virtual Reality (VR) environments [21]. However, this field currently lacks a consistent framework, particularly regarding the quantitative application of Discrete Wavelet Transform (DWT) and the establishment of testing protocols. Even when computational methods are theoretically implemented, the artifacts and measurement errors inherent to various experimental setups frequently compromise the reliability of the data.

Given the increasing volume and complexity of data derived from these multi-modal setups, contemporary research increasingly relies on artificial intelligence (AI) to automate fatigue detection [22]. AI algorithms offer a robust mechanism to combine various signal inputs and identify subtle patterns of exhaustion that traditional statistical methods may overlook [23], and the successful deployment of AI in this context requires a fundamental understanding of the biophysical origin and the artifacts involved [24]. Consequently, substantial potential for advancement lies in the development of hybrid frameworks that integrate advanced signal decomposition (specifically time–frequency analysis) with AI classifiers. Such an approach is paramount for mitigating signal non-stationarity and facilitating the transition from post hoc analysis to continuous, real-time monitoring in dynamic environments.

**Figure 1:**
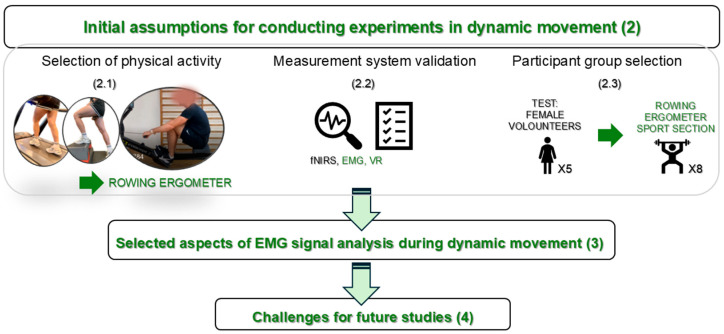
Schematic overview of the structure of the article and experimental workflow.

Based on the authors’ previous work addressing these methodological hurdles [25,26,27], this article presents selected guidance, formulated based on current guidelines and the own experiences gained from research on muscle fatigue in dynamic movement [25,26,27], this article presents selected guidelines, formulated based on current guidelines and the own experiences gained from research on muscle fatigue in dynamic movement. It focuses on the planning process for compiling research results in dynamic movement, addressing difficulties encountered at each stage. This article provides answers to common questions and offers approaches to solving specific problems. Individual issues are discussed based on research conducted on muscle fatigue in a homogeneous group of ergometer athletes. Ultimately, it demonstrates the potential to apply AI algorithms in conjunction with wavelet analysis, specifically highlighting research on fatigue in dynamic movement. Although the authors’ previous work partially addressed the aforementioned issues, this article, informed by current guidelines, is considered to offer valuable insights for planning and executing experiments. The article is organized into sections, as illustrated in the schematic diagram in Figure 1.

The remainder of this article follows the logical progression of the experimental workflow (Figure 1). Section 2 details the foundational study design, covering the rationale for selecting the rowing ergometer task (2.1), the validation of the multimodal measurement system (2.2), and the criteria for participant recruitment of participants (2.3). Subsequent sections address specific methodological strategies for EMG signal analysis during dynamic movement, concluding with a discussion of current limitations and challenges for future research.

Consequently, this manuscript is designed not only as a review of the existing literature but primarily as a methodological proposal and a practical guide. By integrating theoretical foundations with an evaluation of specific technical challenges (e.g., motion artifacts, sensor synchronization) identified during our pilot experiments, this paper aims to provide a robust framework for researchers. It addresses practical instructions for the evaluation of dynamic fatigue to facilitate the design of more reliable experimental setups in future studies.

## 2. Initial Assumptions for Conducting Experiments in Dynamic Movement

Investigating muscle fatigue during dynamic movement demands rigorous experimental planning. The key methodological prerequisites established prior to primary data acquisition include the selection of an appropriate physical activity, the definition of participant inclusion criteria, and the validation of measurement instrumentation. This section details these foundational steps and outlines the process that leads to the optimized research protocol.

### 2.1. Selection of Physical Activity

Selecting physical activity that simultaneously reflects dynamic conditions and allows for controlled repeatable measurement constitutes a key challenge in muscle fatigue research. The preliminary assumptions were based on the identification of the type of experiment and its intended duration. The objective was to select a movement modality that would involve specific muscle groups (in this case, the triceps suprae muscle was the target) and induce fatigue to a subjective maximum and enable fatigue induction to a subjective maximum. Although the current iteration of the protocol focuses on the lower extremities, the rowing ergometer inherently engages the upper body musculature. Consequently, extending the measurement setup to include upper extremity muscles constitutes a natural and planned expansion of this research framework to fully capture compensatory mechanisms during fatigue.

Based on the first work [25], two standardized tests were considered: the Astrand treadmill test, which involves running on a treadmill with gradually increasing incline, and the Astrand–Rhyming step test, which involves stepping onto a step at a set rhythm. One of the purposes of conducting these tests was to check how the measurement equipment would behave (EMG—Ultium EMG (Noraxon) 32 channels, sampling rate 4000 Hz—for recording muscle electrical signals; fNIRS—OctaMon M (Artinis)—measuring changes in muscle oxygenation, two receivers, and eight transmitters using near-infrared light) during long-term dynamic fatigue tests. These tests were chosen due to activation of the triceps suprae muscle and the possibility of assessing maximum fatigue using the Borg scale and variable load.

However, the pilot tests revealed significant methodological limitations. Despite strict adherence to the skin preparation guidelines (specifically, hair removal and thorough cleaning with an alcohol-ether mixture to minimize impedance), electrode displacement and detachment were frequent. Furthermore, the high-impact mechanics inherent in running and stepping introduced substantial motion artifacts into both the sEMG and fNIRS signal streams. These findings required a transition to a dynamic movement task that minimizes measurement disturbances while maintaining biomechanical familiarity for athletes.

Consequently, the use of a rowing ergometer was decided upon. This activity, due to its stationary nature and cyclical movements, facilitates more controlled monitoring of changes in muscle activity and mitigates variability arising from uncontrolled posture and technique, making fatigue measurement more repeatable and reliable. Furthermore, the selection of the ergometer was influenced by the intention to incorporate VR elements into the investigation [28]. Numerous dynamic sports had to be excluded due to the potential health risks to the participants during the measurement, which was conducted in a completely detached environment.

The duration of the experimental trials was not rigidly standardized; instead, the protocol required continuation until the participant reached a state of voluntary exhaustion. This approach was critical, as imposing a fixed time limit would have rendered the results unreliable by failing to capture the true threshold of neuromuscular fatigue. These tests were chosen due to the activation of the specific muscles and the possibility of assessing maximum fatigue using the Borg scale and variable load. Furthermore, in rowing ergometer trials, variable damper resistance and continuous Heart Rate (HR) monitoring were used to safely induce and verify this maximal physiological effort.

### 2.2. Measurement System Validation

During the selection of physical activity, a validation of the measurement instrumentation was carried out to assess its operation and dependability under dynamic conditions. The initial configuration consisted of the EMG Ultium EMG systems (Noraxon), referenced in the previous chapter (2.1), to record muscle electrical signals and the OctaMon M (Artinis) to measure changes in muscle oxygenation (fNIRS). Both were mounted on the triceps suprae muscle, while movement was analyzed concurrently using a camera. Throughout the dynamic tests (treadmill, step), several equipment-related challenges were noted:

- Due to differences in running technique and significant changes associated with leg movement, electrodes are often moved and detached.
- Despite adhering to the literature guidelines, the problem remains unresolved.
- Fatigue tests, although defined by detailed experimental conditions, introduce many artifacts in the readings of both sensors.
- The color of the skin, its thickness and the individual anatomical differences cause disturbances in the fNIRS sensors.
- Synchronizing two independent sensors requires additional time, which affects the quality of research and the well-being of participants.
- The Borg scale provides a good starting point to determine the level of fatigue.

The choice of the rowing ergometer contributed to partial resolution of these issues. Data synchronization between EMG and fNIRS streams was performed manually during the post-processing phase. The application of integrated wearable sensors [29] may eliminate the challenges of manual integration, and this solution may be beneficial in future research to automate the data acquisition process. The more predictable and restricted range of movement on the ergometer reduced the problem of electrode displacement and mitigated the number of movement artifacts in the signals. Furthermore, the controlled indoor laboratory environment inherent to ergometer training minimizes ambient light interference (e.g., direct sunlight), effectively protecting the fNIRS signal from external optical noise. However, it should be noted that the planned expansion of research to include a greater number of sensors, such as an fNIRS unit positioned on the participant’s head, may introduce conflicts with VR goggles, particularly in the context of excessive perspiration during exertion, which has been identified as a potential limitation.

### 2.3. Participant Group Selection

To increase the reliability of the results, particularly in the context of assessing fatigue during effort specific to a given sport discipline, the aim is to study homogeneous groups, such as athletes regularly training a specific discipline. To ensure the reliability of the data, specific measures were implemented to address physiological variability. Inter-personal variability was minimized by recruiting a homogeneous cohort of elite athletes. Furthermore, to mitigate intra-personal variability, participants adhered to a strict preparatory protocol (including dietary and sleep standardization). Detailed descriptions of these inclusion criteria and variability control procedures are provided in our previous experimental works [25,26,27]. One of the most important factors is that the group is selected in a way that ensures that it is representative. Statistical planning prior to the test is also crucial. However, achieving the assumed sample size is often challenging due to the highly demanding nature of research involving dynamic movement. Furthermore, while such homogeneity improves sport-specific analysis, it introduces a potential bias when developing universal AI frameworks, creating the risk of overfitting to the unique biometric patterns of elite athletes. Therefore, these aspects should be carefully considered during the experimental design phase to ensure an appropriate balance between population specificity and model generalizability.

The Borg scale was used to assess the subjective level of fatigue. It constitutes a valuable reference point, allowing for individualization of the test completion time or assessment of fatigue progression.

Psychological and behavioral factors are also significant. The appropriate preparation for the test, the proper hydration of the body, and the motivation of the participants can influence their performance and subjective sensations, which should be considered in the research protocol.

Experimental design should be grounded in a priori statistical power and effect size analysis (e.g., using G*Power 3.1 software), which often indicates a requirement for large sample sizes (N > 50) to maximize statistical power. However, in dynamic research involving elite athletes, such numbers are often logistically unfeasible due to population scarcity. The methodological insights presented in this paper are derived from our previous experimental iterations that involved smaller, highly homogeneous cohorts (e.g., N = 5 to N = 8). Therefore, the focus of this work is to synthesize these experiences into a robust framework that assists researchers in navigating these constraints, prioritizing high data quality and group homogeneity when large-scale recruitment is not possible. From a machine learning perspective, the definition of an adequate sample size can shift from the number of participants to the total number of movement cycles. Through signal segmentation, where each specific motion constitutes a single training instance, even smaller cohorts yield thousands of data vectors. This volume is typically sufficient for feature-based classifiers, while scenarios involving data-demanding Deep Learning models may necessitate the use of Data Augmentation or Transfer Learning techniques. Consequently, the assessment of dataset sufficiency in such frameworks often relies on learning curve analysis rather than classical a priori power estimation.

### 2.4. Summary

**Figure 2:**
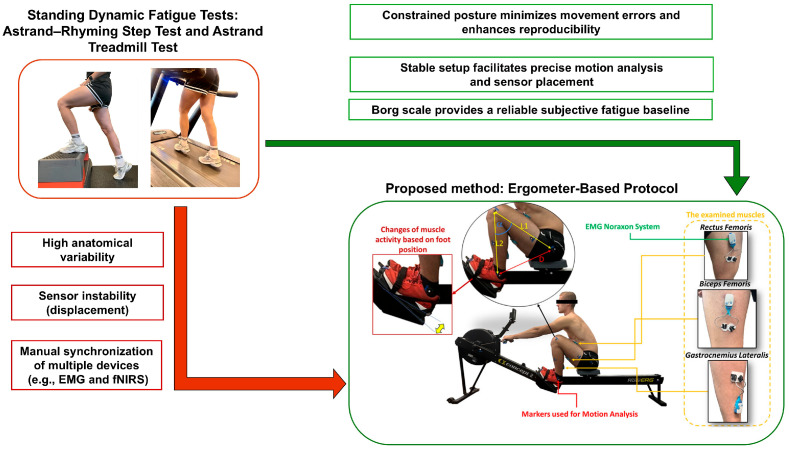
Dynamic fatigue experiments that activate the muscles of the lower extremities are derived from many standing exercises, and the proposed method is based on studies by the previous author [27]—research workflow.

The factors outlined above regarding the selection of physical activity, the validation of the measurement apparatus, and the recruitment of the participant group, informed by the findings of the preliminary studies, established the basis for the development of a novel and detailed measurement protocol. The intent behind these endeavors was to establish the most objective and reproducible conditions feasible for investigating the phenomenon of muscle fatigue in dynamic movement while simultaneously mitigating the impact of confounding factors. The workflow of the research is depicted schematically in Figure 2.

EMG signal analysis is a commonly used technique to study muscle physiology and investigate muscle fatigue [7,30], which provides the ability to monitor changes associated with progressive exertion. The key parameters used in this approach are the MNF and MDF of the EMG signal power spectrum, which are recognized as valuable indicators of fatigue [31,32,33]. Numerous investigations have established that a critical indicator of developing fatigue, observable in spectral analysis, is the typical downward shift in the EMG signal power spectrum toward lower frequencies, which correlates with changes in the conduction velocity of action potentials in muscle fibers. This occurrence is characterized by a systematic decrease in both calculated MDF and MNF values as the duration of fatiguing contractions lengthens [34,35]. A negative linear regression slope coefficient for changes in MDF over time is considered an effective quantitative indicator that allows assessing the onset and progression of muscle fatigue [36,37,38].

The EMG signal is widely considered a non-stationary signal. This means that its general characteristics can change dynamically during the recording of muscle activity. This occurs due to complex physiological phenomena, such as muscle fatigue progression, changes in motor unit recruitment, or fluctuations in the level of generated force. This non-stationarity of the EMG signal is a crucial aspect that must be considered when selecting analysis methods to ensure the reliability and validity of the obtained results. Applying methods that assume stationarity can lead to erroneous interpretations.

## 3. Selected Aspects of EMG Signal Analysis During Dynamic Movement

### 3.1. Standard Spectral Analysis Under Static Conditions

The selection of an appropriate methodology for the analysis of EMG signals, a key factor in muscle fatigue investigations, is dictated by the characteristics of the movement under examination and the attributes of the signal itself. Considering that this analysis, which assesses parameters such as MDF and MNF, is concerned with the relative power distribution in the frequency domain rather than the signal’s absolute amplitude, EMG normalization is not necessary for this kind of evaluation. As highlighted in a review [13], in frequency analysis, the amplitude of the EMG signal is not critical, and normalization of the EMG is not required. Popular analysis techniques include: FFT, Short-Time Fourier Transform (STFT), and WT [31,39,40]. In the case of static (isometric) movement, where the EMG signal can be treated as quasi-stationary in short time windows, it is often sufficient to apply classical methods of spectrum estimation, such as FFT [41], for the calculation and tracking of changes in the parameters described above, such as MDF/MNF. Referring to the previous chapter, the EMG signal exhibits a non-stationary character [42], which can limit the effectiveness of the classical Fourier transform [43], especially during longer analyses or when fatigue increases.

### 3.2. Time–Frequency Analysis for Dynamic Movements

Analysis of dynamic movement, which generates a highly non-stationary EMG signal resulting from complex changes in muscle activity and movement kinematics, necessitates the use of more advanced time–frequency analysis techniques capable of precisely tracking spectral changes over time. For this reason, STFT and WT are often considered to be more appropriate analysis methods under such conditions [44]. STFT analyzes the signal in sliding time windows. However, its use involves a fundamental compromise between temporal and frequency resolution, depending on the width of the chosen analysis window [45]. On the contrary, the wavelet transform is indicated as a method that offers potentially superior accuracy and adaptive time–frequency resolution (better time resolution for high frequencies and better frequency resolution for low frequencies), which is particularly advantageous when analyzing nonstationary signals and dynamic contractions [41,45,46]. Within this family of transforms, the DWT is often preferred over the continuous wavelet transform (CWT) due to its reduced redundancy of information and computational efficiency [43,47]. DWT implementation typically employs filter banks to decompose the signal into approximation and detail coefficients on different frequency scales [47,48], making it an effective tool for EMG analysis in the context of fatigue assessment.

Before proceeding to the subsequent stages of research development and employing additional measurements or computational methods, it is beneficial to familiarize oneself with the differences in calculations between static and dynamic movement. Furthermore, if the research is planned to be extended to include other devices (for example, fNIRS, EEG, or the implementation of VR), it is also necessary to become familiar with the methods of signal filtration, artifact removal, segmentation, and classification.

## 4. Challenges for Future Studies on Fatigue During Dynamic Movement

### 4.1. Integrated Research Framework and Preprocessing

**Figure 3:**
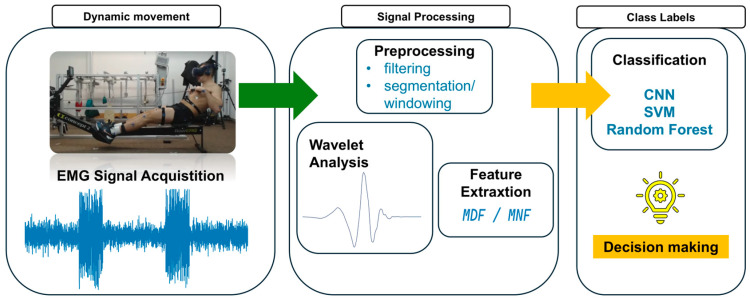
Schematic diagram of the research procedure for fatigue in dynamic movement.

The aspects related to the planning of research on fatigue in dynamic movement, as well as the acquisition and processing of the EMG signal, have been discussed above. These are the main aspects that should be analyzed before beginning research using artificial intelligence tools. Figure 3 presents a schematic diagram of the research procedure for muscle fatigue in dynamic movement.

Within the preliminary processing stage (preprocessing), raw EMG signals are subjected to filtration to eliminate noise and interference outside the physiological frequency band. For this purpose, a Butterworth filter is often employed, configured as a band-pass filter, for instance, with cutoff frequencies of 20 Hz and 500 Hz, which permits the removal of both low-frequency and high-frequency interference. Following the initial preparation of the signal, the primary analysis phase focuses on time–frequency methods, with particular emphasis on WT, often in its discrete form. The efficacy of WT/DWT in analyzing non-stationary EMG signals, particularly those recorded during dynamic movements, justifies its selection. DWT-based analysis involves decomposing the signal into distinct frequency levels using a chosen mother wavelet, such as Daubechies4 (db4), which yields approximation and detail coefficients. Subsequently, the frequency parameters, such as MDF, are calculated from these coefficients or the reconstructed signal, serving as a quantitative measure of changes in muscle fatigue during exercise. Ultimately, the features thus extracted, which condense the information within the EMG signal, are fed into AI algorithms, including both conventional machine learning (ML) methods and deep learning (DL) models. These algorithms undertake tasks such as pattern recognition, classification (e.g., fatigue status), or prediction, leveraging the potential synergy between advanced feature extraction techniques (like WT) and the interpretative capabilities of AI.

AI, particularly ML and DL, enables automatic analysis of complex signals, identification of patterns indicating fatigue that are difficult to recognize, and the construction of predictive models [49]. Various AI/ML/DL algorithms are used in this area; AI models allow the learning of complex patterns from features extracted from EMG data, providing an objective data-driven evaluation of fatigue [7].

Given the scientific need for a quantitative assessment of fatigue in dynamic movement, it was decided to further explore the directions of research on fatigue using AI techniques with wavelet analysis. This focuses on the role of wavelet analysis in feature extraction with the application of AI models (e.g., SVM, CNN, Random Forest) [50,51]. This is intended to define learning and validation strategies, comparing different methodologies and their limitations.

### 4.2. Wavelet-Based Feature Extraction Strategies

For each DWT level, the signal is decomposed into approximation coefficients (cA), which reflect the low-frequency constituents whose amplitude is associated with fatigue development, and detail coefficients (cD), representing the high-frequency constituents. The approximation and detail coefficients themselves, obtained at various levels of the DWT, can serve as feature vectors [52]. These vectors capture the energy distribution of the signal on various time–frequency scales. PCA can be applied to these coefficient vectors for dimensionality reduction [53].

The use of wavelet coefficients primarily serves to segment the EMG signal into more meaningful localized time–frequency components before the application of feature calculations (statistical, spectral, etc.) or their use as input data for machine learning models [52]. The primary advantage lies in the decomposition, rather than the direct use of raw coefficient values in all cases. Many studies extract statistical characteristics from the coefficients [54] or calculate the MDF based on these coefficients [27], rather than using the coefficients themselves as the final characteristic vector. Even when the coefficients are used more directly [53], they represent the energy distribution of the signal in specific bands. This pattern suggests that the primary role of WT here is effective signal decomposition and localization, enabling more significant feature extraction in appropriate sub-bands associated with fatigue.

### 4.3. Optimization of the WT-AI Synergy

The synergy between WT and AI in EMG signal analysis is particularly effective when WT is applied not only for general feature extraction but also as a tool for preliminary signal processing. This encompasses both effective noise removal, which is crucial given the susceptibility of EMG to interference, and the potential enhancement or isolation of specific muscle fatigue biomarkers [52]. For example, advanced wavelet-based denoising techniques, such as improved thresholding methods [49], can help preserve the spectral changes associated with fatigue. Similarly, the decomposition of the signal into precisely selected levels or the application of specific decomposition methods, such as ICEEMDAN [23], allows the isolation of frequencies significant to the fatigue process. Studies achieving the highest classification effectiveness often rely on specific optimizations of the wavelet stage, such as selecting decomposition levels or wavelet coefficients associated with fatigue [52]. This indicates that success in this field is largely due to the skillful use of WT for signal preparation, filtering out interference and highlighting fatigue indicators, which in turn can enable machine learning models to perform more effective and accurate classification. Consequently, numerous studies have demonstrated the effectiveness of an approach in which WT is employed in the preliminary processing stage (denoising) and/or for feature extraction, followed by the AI algorithm performing the classification of the fatigue state.

References can be found in the literature on the utilization of wavelet coefficients or signal energy in specific wavelet bands as input features for classifiers such as SVM or ANN [51]. These results indicate that the optimal AI-WT framework appears to be task-dependent. Different dynamic movements engage different patterns of muscle activation, velocities, and types of contraction. This suggests that tailoring the extraction of WT features and the selection of AI models to the specific biomechanics and anticipated EMG characteristics of a given dynamic task is crucial in muscle fatigue research.

### 4.4. Current State-of-the-Art: A Comparative Review

The ability of wavelet transform to precisely analyze the time–frequency characteristics of non-stationary EMG signals, in combination with the capabilities of AI/ML/DNN in the field of advanced pattern recognition and automatic feature learning, has led to significant progress in this area. A limited number of studies address the topic of fatigue itself, as evident from the above description. However, considering the topic more broadly, not limiting oneself to the dynamic movement aspect alone, the synergistic combination of AI, ML, and deep neural networks (DNN) with WT constitutes a powerful tool in the advanced analysis and processing of EMG signals.

To present the area of scientific research concerning the application of AI/ML/DNN in conjunction with wavelet transform for the analysis and processing of EMG signals, studies presenting selected research methodologies are compiled in Table 1.

This mini-review highlights the main areas of research. They focus primarily on data compression and noise reduction in EMG signals, as well as muscle type classification, hand movement classification, gesture recognition, and EMG signal recognition for prosthetic control. As can be observed, the classification of hand movements and gesture recognition constitutes a significant area of these studies. Research concerning the detection of muscle fatigue and the classification of muscle activity represents a smaller proportion, indicating the need for further development in this thematic area.

## 5. Conclusions

Although the combination of wavelet analysis of EMG signals with machine learning methods represents a promising strategy for detecting muscle fatigue, fully exploiting its potential requires further research focused on several key challenges. A significant limitation of current systems is their limited generalizability beyond specific training conditions, which highlights the need to develop domain adaptation techniques and implement rigorous validation protocols that cover diverse populations and movement tasks. Furthermore, integrating EMG data with information from other physiological sensors, such as NIRS and EEG, within an approach based on multimodal fusion and machine learning, can significantly improve the complexity and reliability of fatigue assessment. Standardization efforts are equally fundamental, including the development of protocols for fatigue induction, statistical methods improvement, data acquisition, criteria for selecting mother wavelets, feature extraction methods and validation, as well as the creation of publicly available reference datasets. Such a harmonization will enable a reliable comparison of the results of different studies and will accelerate the translation of the developed technologies into practical applications. Addressing these challenges is crucial to further progress and the practical implementation of fatigue monitoring systems that use EMG, wavelet analysis, and machine learning. Ultimately, the refinement of this methodology paves the way for applications transcending the realm of competitive sports, finding critical utility in clinical rehabilitation and smart assistive technologies. Such a transition to real-time capability will empower future biofeedback systems to dynamically respond to muscle status, thereby ensuring safety and efficacy in both athletic training and therapeutic recovery.

## Figures and Tables

**Table 1:** Comparative studies on the use of AI methods with wavelet transform.

Source	Technique	AI Methods	Application	Results
[55]	DWT	Intelligent dynamic bit allocation scheme implemented using a Kohonen layer (neural network)	Data compression, noise reduction	It has been demonstrated that the compression performance of the EMG signal is superior compared to standard wavelet algorithms, minimizing distortions at a given compression ratio.
[56]	WT	ANN—MLP and GRNN (input comprises the coefficients of the auto-regressive signal model after WT)	Muscle type classification, feature extraction	The high effectiveness in classifying muscle types was confirmed, and it was demonstrated that preliminary processing of EMG signals using the wavelet transform significantly improves the results.
[57]	DWT	Random Forest, KNN, Decision Tree	Hand movement classification, feature extraction	The effectiveness of different feature extractors and classifiers was compared, identifying combinations that produced good results; DWT features showed competitive effectiveness.
[58]	WPT (wavelet packet transform)	BPNN SVM GA-SVM	Muscle fatigue, muscle activity classification	Identification of muscle fatigue using the GA-SVM classifier, which was more accurate than other approaches.
[59]	DWT	Feed Forward Back Propagation Neural Network (FFBPNN) (ANN)	Hand movement classification, feature extraction	High accuracy was achieved in the classification of hand movements using DWT features and a neural network, indicating the effectiveness of the selected decomposition level.
[60]	CWT	Deep neural networks (ConvNets)	Gesture recognition, Feature extraction (automatic)	Transfer learning was shown to systematically and significantly improve the performance of deep neural networks in EMG gesture recognition, particularly for CWT models.
[61]	DWT	Adaptive Neuro-Fuzzy Inference System (ANFIS).	EMG signal recognition (for prosthetics)	High accuracy in the recognition of EMG signals was achieved, indicating the potential to combine DWT and ANFIS as a control signal for prosthetics.
[62]	DWT	KNN	Recognition of hand movements.	A practical and computationally lightweight multilevel feature extraction method (TP-DWT) was proposed for sEMG signals, which allowed for achieving high accuracy to be achieved in hand movement recognition.
[63]	CWT (cumulative scalograms)	XMANet (Cross-layer Mutual Attention Learning Network) with different CNNs	Gesture recognition, advanced feature extraction	A novel network architecture (XMANet) with attention mechanisms was proposed, which consistently improves the performance of gesture recognition based on CWT scalograms.

## Data Availability

The original contributions presented in this study are included in the article. Further inquiries can be directed to the corresponding author.

## Abbreviations

The following abbreviations are used in this manuscript:

sEMG	Surface Electromyography
WT	Wavelet Transform
fNIRS	Functional Near-Infrared Spectroscopy
EEG	Electroencephalography
AI	Artificial Intelligence
EMG	Electromyography
MDF	Median Frequency
MNF	Mean Frequency
EMD	Empirical Mode Decomposition
HHT	Hilbert–Huang Transform
FFT	Fast Fourier Transform
VR	Virtual Reality
DWT	Discrete Wavelet Transform
CWT	Continuous Wavelet Transform
STFT	Short-Time Fourier Transform
db4	Daubechies 4
ML	Machine Learning
DL	Deep Learning
SVM	Support Vector Machine
CNN	Convolutional Neural Network
PCA	Principal Component Analysis
ANN	Artificial Neural Network
DNN	Deep Neural Network
MLP	Multilayer Perceptron
GRNN	General Regression Neural Network
GA-SVM	Genetic Algorithm-based SVM
FFBPNN	Feed-Forward Back-Propagation Neural Network
ANFIS	Adaptive Neuro-Fuzzy Inference System
KNN	K-Nearest Neighbors
XMANet	Cross-layer Mutual Attention Learning Network
